# A test-based strategy for early return to work for health-care workers with COVID-19 during the Omicron wave, Brunei Darussalam, 2022

**DOI:** 10.5365/wpsar.2024.15.1.1051

**Published:** 2024-02-06

**Authors:** Alice Lai, Ashish Trivedi

**Affiliations:** a Occupational Health Division, Ministry of Health, Brunei Darussalam.; b Pengiran Anak Puteri Rashidah Sa’adatul Bolkiah Institute of Health Sciences, Universiti Brunei Darussalam, Brunei Darussalam.

## Abstract

**Objective:**

This paper summarizes and evaluates a test-based strategy for early return to work for health-care workers (HCWs) with mild coronavirus disease in Brunei Darussalam during the Omicron wave in February 2022 and compares the characteristics of HCWs by how long it took them to return to work.

**Methods:**

The early return-to-work strategy involved testing on day 3 of infection with reverse transcription–polymerase chain reaction and with a rapid antigen test on days 5 and 6 or days 5 and 7. Data about infected HCWs were extracted from the Ministry of Health’s public health surveillance database. Percentages and proportions were used for descriptive statistics, and Pearson’s χ^2^ test and the paired *t*-test were used to compare return-to-work patterns with demographic factors and vaccination status of the HCWs, as well as between cycle threshold (Ct) values and occupational groups of HCWs.

**Results:**

From 15 February to 15 March 2022, a total of 1121 HCWs were notified as being infected with severe acute respiratory syndrome coronavirus 2 (SARS-CoV-2). Of these, 175 (15.6%) were able to return to work on day 4 of their infection, 153 (13.6%) on day 6 and 268 (23.9%) on day 7; 525 (46.8%) required 10 days of home isolation. Statistically significant associations were observed between return-to-work periods and occupational group (*P* < 0.01) and Ct value (*P* < 0.01), but not between return to work and age, sex or vaccination status.

**Discussion:**

This test-based strategy ensured a balance between mitigating a shortage of HCWs and enabling them to return to work early without compromising their safety and that of their patients.

Since the start of the coronavirus disease (COVID-19) pandemic in early 2020, many countries have faced either their third or fourth wave of the outbreak, mainly due to new variants and subvariants of severe acute respiratory syndrome coronavirus 2

(SARS-CoV-2). Health-care workers (HCWs), therefore, were at the highest risk for COVID-19 as a direct consequence of their occupational exposure to the virus. (*1*) With the health-care sector experiencing staffing shortages as a result of the increasing number of cases occurring with each wave, health-care facilities faced challenges in managing the pandemic while maintaining essential health services. (*1*) This burden was further compounded by the absence of HCWs due to them becoming infected with SARS-CoV-2, experiencing the psychological effects of the pandemic, being unable to attend work if they were the main caregivers for infected and ill family members, and being in quarantine or self-isolation as a result of close contact with someone with COVID-19. (*2*, *3*)

Health services required additional staffing during the pandemic to maintain appropriate functioning but still had to consider how to maintain a safe work environment for HCWs. (*4*) During the pandemic, several strategies were implemented by countries to avoid shortages of essential HCWs. This included hiring additional staff, limiting non-essential health services, restricting non-essential annual leave, implementing early return-to-work (RTW) policies for HCWs with COVID-19 and enforcing strict workplace surveillance for asymptomatic HCWs who are in close contact with patients confirmed or suspected to have COVID-19. (*4*-*6*)

Following the first case of COVID-19 in Brunei Darussalam on 9 March 2020, the country had three waves of outbreaks. There were 337 cases during the first wave, from 9 March to 31 July 2021; 16 139 cases during the second wave (Delta variant), from 1 August 2021 to 31 January 2022; and 124 066 cases during the third wave (Omicron variant), from 1 February to 20 April 2022, at the time of this report. (*7*-*9*) There was no confirmed local transmission to HCWs during the first wave; however, during the second wave, 394 HCWs were infected. (*10*) The number increased significantly during the third wave, such that in the first 2 weeks of the third wave, in February 2022, 474 HCWs were infected. This number rose to 2345 infected HCWs by 20 April 2022.

The Ministry of Health (health ministry) saw a need to step up mitigation measures to detect cases early and to break the onward chain of transmission. (*10*) One of these was a test-based early RTW strategy for HCWs. During the first and second waves, HCWs with COVID-19 followed the same testing and isolation protocol as the community. However, due to the significant number of HCWs affected during the third wave, a revised strategy was implemented for HCWs beginning on 15 February 2022 (**Fig. 1**). The revised HCW protocol was circulated to all health-care facilities through the heads of departments or services and supervisors. Infected HCWs were to undergo reverse transcription–polymerase chain reaction (RT–PCR) testing on day 3 of infection, and if they were negative or positive with a Ct value of ≥ 30, they could end isolation and return to work by day 4. If their Ct value was < 30 on day 3, they needed to continue isolation and perform exit tests as per the community health protocol.

**Fig. 1:**
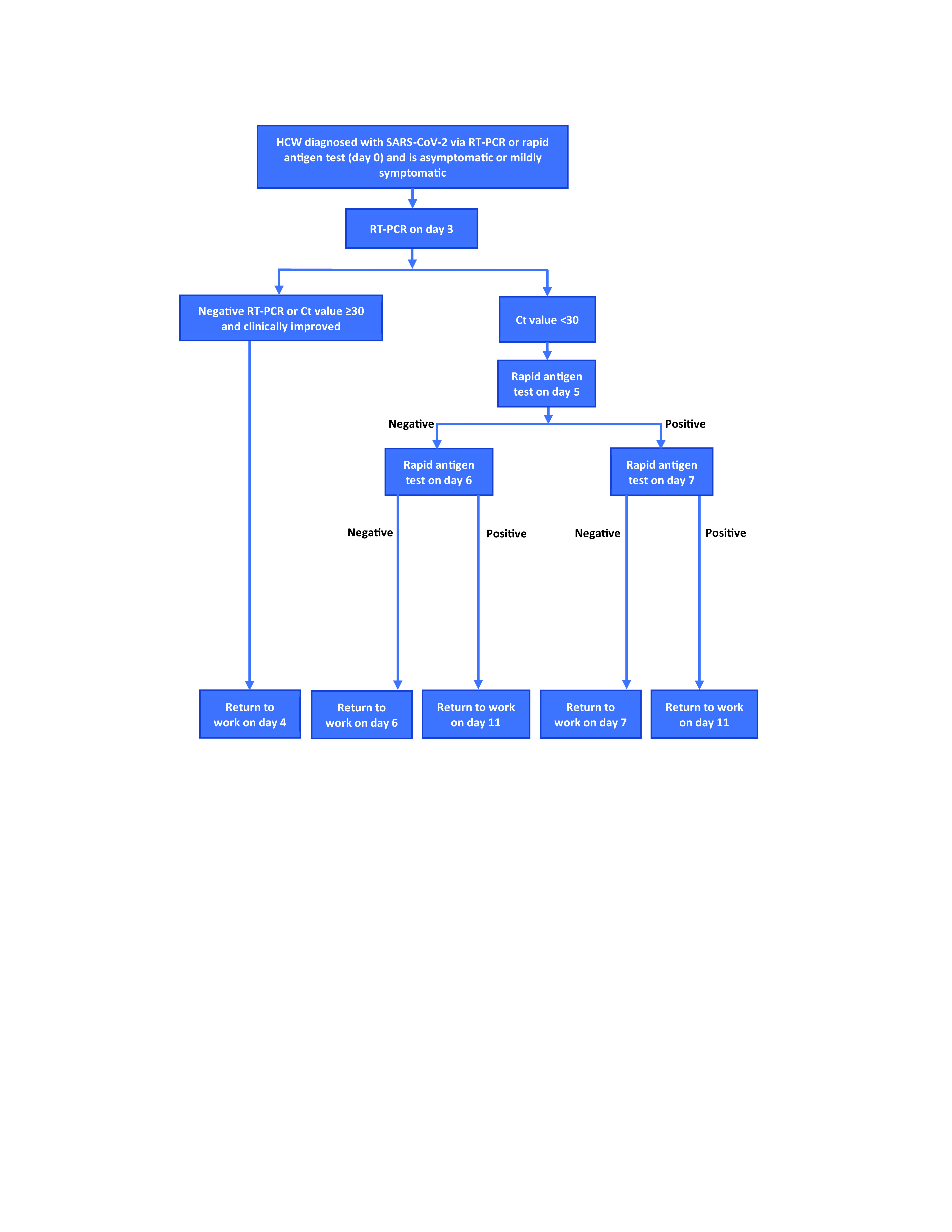
Flowchart of the test-based strategy for early return to work for health-care workers, Brunei Darussalam, effective 15 February 2022

[**insert** Figure 1]

The third-wave community health protocol required infected individuals to undergo mandatory home self-isolation for a minimum of 6 days or a maximum of 10 days, depending on the outcome of their exit tests. If the individual had two consecutive negative rapid antigen test results on days 5 and 6, they could end isolation. If they were positive on day 5, they took another test on day 7. If they were negative on day 7, they could end isolation. If their day 6 or day 7 result was positive, they needed to complete 10 days of isolation.

To ensure compliance with self-testing using the rapid antigen test during home isolation, results from the test were uploaded onto the health ministry web portal via the BruHealth mobile application, a one-stop mobile platform used for contact tracing and identifying positive cases of COVID-19 in Brunei Darussalam. This electronic platform also featured access to a self-assessment health tool, entry and exit QR code to be scanned for accessing public premises, access to online personal health records and updates on the national and global situation of the COVID-19 pandemic, among others. (*11*, *12*) On uploading their negative exit test result or completion of 10 days of isolation, the BruHealth code would change colour from purple (indicating a positive case and therefore barring the individual from entering public premises) to green (indicating the individual was negative for COVID-19 and had no underlying medical conditions) or yellow (indicating the individual was negative for COVID-19 but had underlying chronic medical conditions).

The objective of this study is to summarize the outcomes of the test-based early RTW strategy of HCWs in Brunei Darussalam and compare the characteristics of the HCWs by their RTW period.

## Methods

Data about infected HCWs were extracted from the health ministry public health surveillance database. This national database was updated daily and contained data about HCWs with COVID-19 who had been diagnosed by RT–PCR or rapid antigen testing. Data on HCWs who were diagnosed from 15 February to 15 March 2022 were analysed until the end of their isolation period.

For infected HCWs, testing by RT–PCR on day 3 was carried out at any health ministry-designated swab facility; health ministry-approved kits for self-testing with the rapid antigen test were distributed through a coordinated, multisectoral COVID-19 relief agency.

Data were analysed using Epi Info version 7.2.0.1 (Centers for Disease Control and Prevention, Atlanta, GA, USA). Percentages and proportions were used for descriptive statistics, and Pearson’s χ^2^ test and the paired *t*-test were used to compare RTW patterns with demographic factors and vaccination status, as well as Ct values and occupational groups of HCWs.

## Results

**Fig. 2:**
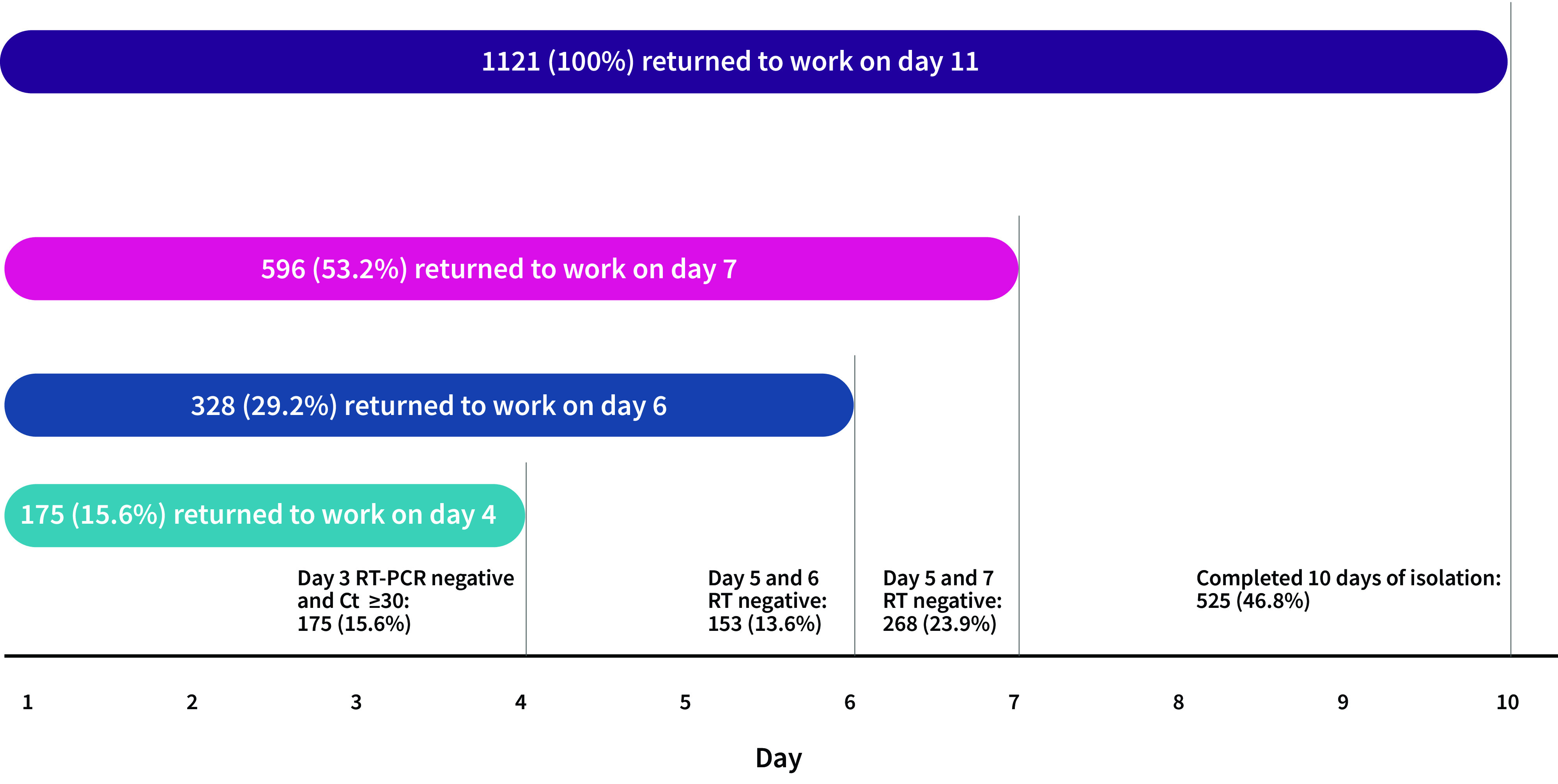
Number of health-care workers with COVID-19, by the day they returned to work, Brunei Darussalam, 15 February to 15 March 2022

A total of 1643 HCWs from government and private health-care facilities were diagnosed with COVID-19 during the study period. Of these, 522 were excluded from the study due to missing information for the day 3 RT–PCR test or the day 5, 6 or 7 rapid antigen test. Of the 1121 infected HCWs included, 139 (12.4%) had a negative RT–PCR result and 36 (3.2%) had a Ct value of ≥ 30 on their day 3 test. Therefore, 175 (15.6%) HCWs were able to return to work after 3 days of isolation (**Fig. 2**). A further 153 (13.6%) were able to return to work on day 6 and 268 (23.9%) on day 7. The remaining 525 (46.8%) HCWs were required to complete 10 days of isolation.

[**insert** Figure 2]

The majority of infected HCWs were female (68.1%, 763), and more than one third were in the 31–40-year age group (34.3%, 384), with a mean age of 38 ± 12 years (**Table 1**). Nurses were the occupational group most affected (45.9%, 514), followed by support staff (i.e. health technicians, porters, attendants, voluntary health workers, at 21% [235]) and allied health professionals (i.e. radiographers, physiotherapists and occupational therapists, optometrists and laboratory staff, at 10.8% [121]).

**Table 1:** Demographic characteristics, cycle threshold values and vaccination status of health-care workers with COVID-19, by day of return to work, Brunei Darussalam, 15 February to 15 March 2022 (*n* = 1121)

Characteristic	Day returned to work^a^				Total	*P* ^b^
	4	6	7	11		
Age group (years)						
≤ 20	3 (27.3)	3 (27.3)	1 (9.1)	4 (36.3)	11 (1.0)	0.30
21–30	46 (15.0)	49 (15.9)	72 (23.5)	140 (45.6)	307 (27.4)	
31–40	63 (16.4)	45 (11.7)	80 (20.8)	196 (51.1)	384 (34.3)	
41–50	42 (16.4)	33 (12.9)	73 (28.5)	108 (42.2)	256 (22.8)	
> 50	21 (12.9)	23 (14.1)	42 (25.8)	77 (47.2)	163 (14.5)	
Sex						
Female	128 (16.8)	108 (14.1)	179 (23.5)	348 (45.6)	763 (68.1)	0.32
Male	47 (13.1)	45 (12.6)	89 (24.9)	177 (49.4)	358 (31.9)	
Occupational group						
Medical practitioner	16 (18.2)	8 (9.1)	6 (6.8)	58 (65.9)	88 (7.9)	< 0.01
Nursing staff	73 (14.2)	66 (12.8)	126 (24.5)	249 (48.4)	514 (45.9)	
Paramedic staff	7 (20.0)	4 (11.4)	9 (25.7)	15 (42.9)	35 (3.1)	
Dental practitioner or staff	9 (27.3)	4 (12.1)	9 (27.3)	11 (33.3)	33 (2.9)	
Allied health professional	18 (14.8)	11 (9.1)	29 (24.0)	63 (52.1)	121 (10.8)	
Administrative staff	7 (9.5)	16 (21.6)	25 (33.8)	26 (35.1)	74 (6.6)	
Support staff	42 (17.8)	40 (17.0)	57 (24.3)	96 (40.9)	235 (21.0)	
Security staff and cleaners	3 (14.3)	4 (19.0)	7 (33.3)	7 (33.3)	21 (1.9)	
Results of diagnostic test						
RT–PCR cycle threshold value						
≥ 30	123 (49)	8 (3.2)	26 (10.4)	94 (37.6)	251 (22.4)	< 0.01
21–30	32 (11.7)	35 (12.8)	70 (25.6)	136 (49.8)	273 (24.4)	
11–20	14 (3.1)	85 (19.0)	133 (29.8)	215 (48.1)	447 (39.8)	
Rapid antigen test positive	6 (4.0)^c^	25 (16.7)	39 (26.0)	80 (53.3)	150 (13.4)	
Vaccination status						
Complete^d^	14 (18.2)	15 (19.5)	20 (25.9)	28 (36.4)	77 (6.9)	0.15
Complete plus booster^e^	160 (15.4)	137 (13.1)	248 (23.8)	497 (47.7)	1042 (93.0)	
Incomplete	1 (50.0)	1 (50.0)	0	0	2 (0.2)	
Booster (*n* = 1 042)						
Within < 1 month	11 (27.5)	4 (10.0)	8 (20.0)	17 (42.5)	40 (3.8)	0.13
Within 1–3 months	72 (16.7)	56 (13.0)	91 (21.1)	213 (49.2)	432 (41.5)	
Within > 3 months	76 (13.3)	76 (13.3)	150 (26.3)	268 (47.1)	570 (54.7)	

RT–PCR: reverse transcription–polymerase chain reaction.

^a^ Values are number (%).

^b^ *P*-values were calculated using Pearson’s χ^2^ test.

^c^ These are health-care workers who underwent rapid antigen testing instead of RT–PCR.

^d^ This refers to having completed two doses of a WHO-approved COVID-19 vaccine.

^e^ A booster is an additional dose beyond the primary two-dose series of a WHO-approved COVID-19 vaccine.

Statistically significant associations were observed between RTW periods and occupational group (*P* < 0.01) and Ct value (*P* < 0.01); however, there were no significant associations between RTW periods and age, sex or vaccination status. A higher proportion of HCWs with direct clinical contact – such as medical practitioners (65.9%, 58), allied health professionals (52.1%, 63), nursing staff (48.4%, 249), paramedic staff (42.9%, 15) and support staff (40.9%, 96) – had a positive result on day 7 and completed 10 days of isolation compared with those who had indirect or no clinical contact with patients. Similarly, a significant association was observed between RTW patterns and baseline Ct values: 49% (123) of HCWs with high baseline Ct values of ≥ 30 were able to return to work by day 4, while those with Ct values < 30 spent longer in isolation (*P* < 0.01).

At the time of diagnosis, 93% (1042) had received three doses of COVID-19 vaccine, while 6.9% (77) had received two doses. Among those who had three doses, 54.7% (570) had their third dose more than 3 months before infection, while 41.5% (432) had theirs 1–3 months prior and 3.8% (40) had theirs within 1 month before infection. There was no significant difference between RTW pattern and vaccination status (whether the HCW had two or three doses) or the booster period (i.e. the time between the third dose of COVID-19 vaccine and COVID-19 diagnosis) (**Table 1**).

In a comparison of Ct values at baseline and at day 3, 83.4% (116/139) of HCWs who had a high Ct value of ≥ 30 at diagnosis transitioned to a negative RT–PCR result by day 3. There were no significant associations between vaccination status and booster period and a change in Ct value from baseline to day 3 (**Table 2**).

**Table 2:** Change in cycle threshold values from baseline to day 3 and association of the change with vaccination status for health-care workers with COVID-19, Brunei Darussalam, 15 February to 15 March 2022

Change in Ct values	No. of HCWs	Ct value at day 3^a^			
		10–20	21–30	> 30	Negative
Ct value at day 0					
10–20	447	154 (34.5)	279 (62.4)	9 (2.0)	5 (1.1)
21–30	273	132 (48.4)	109 (39.9)	19 (7.0)	13 (4.8)
≥ 30	251	87 (34.7)	41 (16.3)	7 (2.8)	116 (46.2)
Rapid antigen test positive	150	71 (47.3)	73 (48.6)	1 (0.7)	5 (3.3)
Total	1121	444	502	36	139
**Change in Ct values and** **association with vaccination**	**No. of HCWs**	**Ct value^b^**			***P* ^c^**
		**Day 0**		**Day 3**	
Total	975	20.9 (12.9)		21.5 (7.3)	0.74
Vaccination status					
2 doses	54	22.1 (11.3)		22.35 (8.3)	0.48
3 doses	921	20.9 (12.9)		21.5 (7.1)	0.56
Booster period					
Within < 1 month	36	22.5 (15.9)		24.7 (11.8)	0.74
Within 1–3 months	393	21.3 (14.4)		21.9 (7.5)	0.85
Within > 3 months	546	20.7 (11.9)		20.9 (6.9)	0.41

Ct: cycle threshold; HCW: health-care worker.

^a^ Values are number (%).

^b^ Values are median (interquartile range).

^c^ *P*-values were calculated using the paired *t*-test.

## Discussion

The adoption of a test-based strategy incorporating RT–PCR testing on day 3 for HCWs with asymptomatic or mild COVID-19 infection enabled essential HCWs to return to work safely and with minimal risk of disease transmission to their patients. This also mitigated issues of staffing shortages for HCWs. Altogether, 15.6% of infected HCWs cleared high infectivity levels by their day 3 RT–PCR testing and, therefore, were able to return to work early. More than half (53%) of the HCWs were deemed safe to return to work after day 7, whereas 47% still had a positive rapid antigen test result at day 7 and required a longer duration of isolation. A study in the United States of America reported a similar proportion of HCWs (43%) with a positive result on rapid antigen testing from day 5 to day 10 during the Omicron wave (Landon E, Bartlett AH, Marrs R, Guenette C, Weber SG, Mina MJ, University of Chicago, unpublished data, 2022.

Our findings showed a significant association between a HCW’s occupational group and RTW period in that HCWs who had direct clinical contact (high-risk HCWs) took longer to recover from COVID-19 compared with those who had indirect (moderate-risk HCWs) or no clinical contact (low-risk HCWs). This can be attributed to an increased risk of disease transmission in the high-risk occupational groups who were managing COVID-19 cases or suspected cases in a hospital setting, treating cases with influenza-like illness in outpatient clinics or performing RT–PCR testing at swab centres, and who had more frequent surveillance testing for SARS-CoV-2. This surveillance testing occurred thrice weekly and comprised one RT–PCR test and two rapid antigen tests for HCWs who were at high risk of infection, compared with the protocol for those at moderate risk, which was RT–PCR testing twice a month and rapid antigen testing twice a week, and the protocol for those considered to be at low risk, which was RT–PCR testing once a month and rapid antigen testing once a week. (*13*) This testing strategy allowed for early detection of COVID-19 in presymptomatic HCWs, which subsequently also resulted in a longer period of isolation. A similar finding was observed in a study in the United States of America, in which positivity on rapid antigen testing and a longer duration of isolation were reported among frequently screened university students compared with infrequently screened groups. (*14*)

Our study also reported an early RTW pattern among a substantial proportion of HCWs (49%) who had Ct values of ≥ 30 at diagnosis. This could have been due to the virus being detected at a later stage of infection, particularly among the low-risk group of HCWs who underwent less stringent regular surveillance and SARS-CoV-2 testing. (*13*) No significant association was observed between the RTW pattern and vaccination status or booster period after primary vaccination. Similarly, no association was seen between vaccination status or booster period and Ct value on day 0 and day 3. This is similar to findings from two studies in the United States of America that looked at HCWs and university students during the Omicron wave, whereby primary COVID-19 vaccination did not have any protective effect on rapid antigen test positivity beyond day 5, and boosted individuals needed a longer duration of isolation (Landon E, Bartlett AH, Marrs R, Guenette C, Weber SG, Mina MJ, University of Chicago, unpublished data, 2022). (*14*)

In conclusion, the introduction of RT–PCR testing on day 3 resulted in 15.6% of HCWs being able to return to work by day 4. Although this proportion may appear low, it had a significant and positive impact on the health workforce crisis during the pandemic when every contribution by a HCW was most welcome. Such a test-based RTW strategy also helped maintain a balance between infection prevention and control measures and mitigation of staff shortages, particularly during the Omicron wave, which saw higher transmissibility and immunity evasion properties of the virus, and resulted in a large number of HCWs becoming infected as a result of occupational exposure and community exposure.

## References

[1] Health workforce policy and management in the context of the COVID-19 pandemic response: interim guidance, 3 December 2020. Geneva: World Health Organization; 2020. Available from: https://apps.who.int/iris/handle/10665/337333, accessed 4 March 2023.

[2] Mascha EJ, Schober P, Schefold JC, Stueber F, Luedi MM. Staffing with disease-based epidemiologic indices may reduce shortage of intensive care unit staff during the COVID-19 pandemic. Anesth Analg. 2020 Jul;131(1):24–30. 10.1213/ANE.0000000000004849 32343514 PMC7173088

[3] Poortaghi S, Shahmari M, Ghobadi A. Exploring nursing managers’ perceptions of nursing workforce management during the outbreak of COVID-19: a content analysis study. BMC Nurs. 2021 Jan 29;20(1):27. 10.1186/s12912-021-00546-x 33514351 PMC7844784

[4] Ruscetti A, Chrisman M, Wagester S, Smith P, O’Hare C, Mallon A, et al. Healthcare personnel early return-to-work program after higher-risk SARS-CoV-2 exposure: A learning health system quality improvement project. Am J Infect Control. 2022 May;50(5):542–7. 10.1016/j.ajic.2022.01.027 35131348 PMC8813718

[5] Zhang JC, Findlater A, Cram P, Adisesh A. Return to work for healthcare workers with confirmed COVID-19 infection. Occup Med (Lond). 2020 Jul 17;70(5):345–6. 10.1093/occmed/kqaa092 32432325 PMC7313860

[6] Strategies to mitigate healthcare personnel staffing shortages [website]. Atlanta (GA): United States Centers for Disease Control and Prevention; 2022. Available from: https://www.cdc.gov/coronavirus/2019-ncov/hcp/mitigating-staff-shortages.html, accessed 4 March 2023.

[7] One new case COVID-19 reported today, 31 July 2021: press release on the current situation of the COVID-19 infection in Brunei Darussalam. Bandar Seri Begawan: Ministry of Health, Brunei Darussalam; 2021. Available from: https://www.moh.gov.bn/Lists/Latest%20news/NewDispForm.aspx?ID=970&ContentTypeId=0x0104009A3003A09F8D6E42981D262E322516A2, accessed 4 March 2023.

[8] 63 new cases COVID-19 reported today, 31 January 2022: media statement on the current situation of COVID-19 in Brunei Darussalam. Bandar Seri Begawan: Ministry of Health, Brunei Darussalam; 2022. Available from: https://www.moh.gov.bn/Lists/Latest%20news/NewDispForm.aspx?ID=1157&ContentTypeId=0x0104009A3003A09F8D6E42981D262E322516A2, accessed 4 March 2023.

[9] 178 new cases COVID-19 reported today, 20 April 2022: media statement on the current situation of COVID-19 in Brunei Darussalam. Bandar Seri Begawan: Ministry of Health, Brunei Darussalam; 2022. Available from: https://www.moh.gov.bn/Lists/Latest%20news/NewDispForm.aspx?ID=1223&ContentTypeId=0x0104009A3003A09F8D6E42981D262E322516A2, accessed 4 March 2023.

[10] Trivedi A, Fontelera M, Lai A. SARS-CoV-2 screening of health care workers in Brunei Darussalam. Workplace Health Saf. 2022 Oct;70(10):452–8. 10.1177/21650799211062802 35112612

[11] Guidelines for healthcare workers confirmed positive for SARS-CoV-2, 14 Feb 2022. Bandar Seri Begawan. Brunei Darussalam: Ministry of Health; 2022.

[12] BruHealth. Bandar Seri Begawan: Ministry of Health, Brunei Darussalam; 2022. Available from: https://www.moh.gov.bn/SitePages/bruhealth.aspx, accessed 23 April 2022.

[13] Healthcare workers’ COVID-19 surveillance strategy in Brunei Darussalam – endemic phase, as of 13 February 2022. Bandar Seri Begawan. Brunei Darussalam: Ministry of Health; 2022.

[14] Earnest R, Chen C, Chaguza C, Hahn AM, Grubaugh ND, Wilson MS; Yale COVID-19 Resulting and Isolation Team. Daily rapid antigen testing to tailor university COVID-19 isolation policy. Emerg Infect Dis. 2022 Dec;28(12):2455–62. 10.3201/eid2812.220969 36417936 PMC9707582

